# The role of systemic statins in the inception and healing of apical periodontitis: a systematic review

**DOI:** 10.1186/s12903-023-03472-3

**Published:** 2023-10-07

**Authors:** Francesca Ideo, Matteo Francesco Manca, Sadia Niazi, Francesco Mannocci, Giulia Bardini, Elisabetta Cotti

**Affiliations:** 1 Department of Conservative Dentistry and Endodontics, University of Cagliari, Cagliari, Italy; 2 Department of Endodontology, Centre for Oral, Clinical & Translational Sciences, Faculty of Dentistry, Oral and Craniofacial Sciences, King’s College London, Guy’s Hospital, London, UK

**Keywords:** Apical periodontits, Systemic statins, Endodontic outcome

## Abstract

**Objectives:**

Statins are a category of medications widely used to reduce plasma LDL-cholesterol levels, that also possess antibacterial, anti-inflammatory, and immunomodulatory action.

The aim of this systematic review was to explore the effects of systemic statins therapy on the development and treatment of apical periodontitis (AP) on humans and animals.

**Material and methods:**

Three electronic databases (PubMed, Web of Science, and Scopus) and grey literature were searched from their inception until February, 20 2023 (PROSPERO CRD42021246231). For the quality assessment and risk of bias, different guidelines were used according to the typology of the studies considered (Animal Research Reporting of In Vivo Experiments, Newcastle–Ottawa Quality Assessment Form for Cohort Studies, Systematic Review Centre for Laboratory animal Experimentation Risk of Bias tool and Tool to assess risk of bias in cohort studies of CLARITY Group).

**Results:**

Seven hundred eleven records were screened, and six articles were included for this qualitative review. The eligible studies showed a moderate overall quality and risk of bias. Human patients in treatment with statins exhibited a higher healing rate of AP following root canal treatment. In experimental animal models, statins had a beneficial effect on the development of AP.

**Conclusions:**

Despite the limited number of studies and considering that most of them are on animals, our findings suggest that systemically administered statins make a positive contribution to prevent the development and help healing of AP.

**Clinical relevance:**

There is an increased evidence that a pharmacologic adjunct to endodontic treatment may be considered to enhance healing of AP. Among other medications, statins seem to have a positive impact on the disease.

**Supplementary Information:**

The online version contains supplementary material available at 10.1186/s12903-023-03472-3.

## Introduction

Apical periodontitis (AP) is an inflammatory disease characterized by the host reaction to an infection caused by polymicrobial colonization in the root canal [1–3]. One of the pathognomonic signs of AP is the presence of an osteolytic lesion within the maxillary bones [4]. Non-surgical root canal therapy (NsRCT) is the treatment of choice for AP and is used to eliminate or reduce the bacterial load inside the endodontium, thus resolving the inflammatory reaction and promoting healing of the periapical tissues [5].

Approximately, in 75–80% of cases, AP heals after NsRCT [6]. The persistence of the disease is often associated with a lack of adequate instrumentation or disinfection of the root canal system [7], but it has also been emphasized that the host immune system plays a role in the outcome of endodontic treatment [8, 9]. Considering that the inception, clinical manifestations, and post-treatment healing response of AP are influenced by the intensity and duration of the inflammatory response, as well as by the velocity of its resolution [10–13], it is reasonable to reflect on the benefit of a pharmacological adjunct to a well performed endodontic treatment of AP [13–20]. Therapeutics that modulate the patient’s immune response either by limiting the intensity of the inflammation, or by promoting the return to homeostasis may contribute to the healing of AP following a correct therapy. Whether these agents are locally (i.e., in the form of intracanal medications) [13–15] or systemically administered [15–20], they may improve the outcome of endodontic treatment.

Among these medications, a possible beneficial effect of the use of statins on the inception and healing of AP has been suggested by recent endodontic literature [18, 21–26].

Statins are the most effective drugs for reducing plasma LDL-cholesterol levels [27]. They act by competitive inhibition of 3-hydroxy-3-methylglutaryl-coenzyme A reductase (HMGCoA-reductase), the regulatory enzyme involved in the synthesis of cholesterol, which diminishes the intracellular concentration of cholesterol, increases the number of HDL receptors on the cell surface and the uptake of plasma LDL by hepatocytes, leading to a decrease in cholesterolemia [28]. Moreover, several experimental studies have suggested that statins exhibit a larger spectrum of action, defined as the *pleiotropic* effects [29–32] The potential of these drugs is linked to the inhibition of the synthesis of isoprenoids (intermediates of the cholesterol biosynthetic pathway), also derived from HMGCoA activity [29, 31]. The result is the post-translational prenylation of small GTP-binding proteins such as Rho and Rac and inhibition of their effectors, Rho kinase and nicotinamide adeninedinucleotide phosphate oxidases [30, 31]. In cell culture and animal studies, these effects alter the development of cardiac hypertrophy and fibrosis, stability of atherosclerotic plaques, expression of endothelial nitric oxide synthase, and production of pro-inflammatory cytokines and reactive oxygen species [29, 32].

In dental field, administration of statins has shown a favorable effect in preventing alveolar bone loss in animal experimental periodontitis [33], while humans with chronic periodontitis and on statins, exhibited less tooth loss [34], and lower signs of periodontal lesions, compared to controls [35]. Furthermore, hyperlipidemic patients taking statins had less gingival bleeding and probing compared to not statin users [36]. Most importantly, a randomized clinical trial concluded that statins significantly reduced the periodontal inflammation in a dose-dependent manner [37].

Therefore, this systematic review aimed to identify relevant clinical studies that have examined the relationship between the systemic administration of statins and the incidence, prevalence, and/or healing of AP.

## Materials and methods

The current systematic review was conducted and reported according to the Preferred Reporting Items for Systematic Reviews and Meta-Analyses Protocols statement 2020 (PRISMA statement 2020) [38, 39] (Supplementary Table 1, Supplementary table 2). The protocol was defined and agreed to by all authors, and it was registered under the International Prospective Register of Systematic Reviews – PROSPERO (registration number CRD42021246231) [40].

### Focused question

*Can systemic administration of statins affect the inception and healing of AP in humans and animal models?*

#### Inclusion criteria

Based on the PICOS [41], the main criteria for considering studies for this review are as follows:

- Types of participants: patients with an AP.
- Types of interventions (test group): studies evaluating the effect of the only systemic administration of statins on inception, development and healing of AP.
- Comparison (control group): patients with AP not in treatment with statins.
- Outcome: prevalence and healing of AP.

Studies where participants do not present at least one AP, ex vivo, in vitro*, and *in silico* models only* studies, studies that included experimental models with comorbidities or with administration of other medications, studies that did not provide a detailed description of the methodology used, review papers, opinion articles and conference abstracts were excluded.

### Literature search strategies

The search was conducted independently by two reviewers (FI and MFM). The following electronic databases were searched: PubMed (https://www.ncbi.nlm.nih.gov/pubmed), Scopus (https://www.scopus.com), and Web of Science (https://www.webofscience.com/wos/woscc/basic-search) from the date of foundation of the databases to February, 20 2023. Supplemental research was performed by screening the reference sections of the relevant studies eligible for inclusion in the present systematic review. Furthermore, some digital repositories available such as Google Scholar™ (first one hundred results were considered) and OpenGrey (http://www.opengrey.eu) were investigated in order to explore any conference papers, unpublished data and grey publications. In addition, other resources, including the citations and reference lists of relevant articles and textbooks, were searched manually.

The following keywords were used for research:

- (statin OR simvastatin OR statins) AND (apical periodontitis OR endodontic treatment OR endodontic lesion OR endodontic OR endodontics OR root canal treatment OR endodontic diseases OR periapical lesion OR pulp healing). Details of the number of articles retrieved from each database are shown in Supplementary table 3.

### Study selection

Articles found from the above search strategy, after removing duplicates, were first screened by two calibrated reviewers (FI and MFM) based on the relevance of the title and abstract, and sequentially excluded according to the eligibility criteria. Prior to the formal screening process, a calibration exercise was undertaken to pilot and refine the screening questions. To obtain an agreement between the authors, 10% of the publications were randomly selected and their classification was compared, and then a Kappa statistic was determined (Kappa = 0.80). During the second screening, full-text articles were reviewed to make the final inclusion/exclusion decision. In cases of disagreement, a consensus was obtained through discussion or by involving a third reviewer (EC). Articles that fulfilled all the criteria after reading the full text were selected for detailed data processing.

### Quality assessment

Relevant appraisal tools mentioned in the literature were used to classify the level of information in the articles and assess the risk of bias. The quality appraisal checklists were elaborated on a system where each item was assigned 0 if the item was not fulfilled, 0.5 if partially fulfilled, or 1 if completely fulfilled, and scored independently by two reviewers (FI and MFM). The differences were resolved through discussion. In cases of disagreement, a third reviewer (EC) was involved for reaching a consensus.

Animal studies were evaluated according to the Animal Research Reporting of In Vivo Experiments (ARRIVE) guidelines [42]. Individual full texts were assessed; articles scoring ≤ 5 were classified as low-level, articles with a score between 5 and 15 as moderate, and articles with a score ≥ 15 as high-level information.

The only cohort study included was evaluated using the “Newcastle–Ottawa Quality Assessment Form for Cohort Studies” guidelines [43]. In the evaluation of the full texts, articles with scores less than or equal to 2 were classified as low-level information, scores between 2 and 7 as moderate-level, scores greater than or equal to 7 as high-level information.

### Risk of bias

The Systematic Review Center for Laboratory Animal Experimentation (SYRCLE) RoB tool was used to assess the risk of bias in animal intervention studies [44]. Eligible articles were evaluated and studies scoring ≤ 3 were classified as having a high risk of bias, those scoring between 3 and 7 as having a moderate risk of bias, and those scoring ≥ 7 as having a low risk of bias.

The “Tool to assess risk of bias in cohort studies of CLARITY Group” was used to grade the risk of bias for the only cohort study included (https://www.evidencepartners.com/wp-content/uploads/2017/09/Tool-to-Assess-Risk-of-Bias-in-Cohort-Studies.pdf). Among the eligible articles, those scoring ≤ 2 were classified as high risk, those scoring between 2.5 and 6.5 as moderate risk, and those scoring ≥ 7 as low risk of bias.

### Data extraction and synthesis

Data extraction was carried out independently by two reviewers (MFM and FI), using a specially designed form, and the accuracy of the data collected was confirmed by a third reviewer (EC). The following information was extracted: title of the article, authors and year of publication, animal species, samples, disease induction, statins considered, statin mode of administration, treatment period, follow-up, outcome measurements and methods, results and source of funding.

Substantial heterogeneity of methods emerged from the selection of the studies, which precludes the conduct of quantitative data synthesis necessary for a meta-analysis. The clinical results were extracted and qualitatively summarized and a descriptive systematic review was performed.

## Results

### Studies selection

**Fig. 1:**
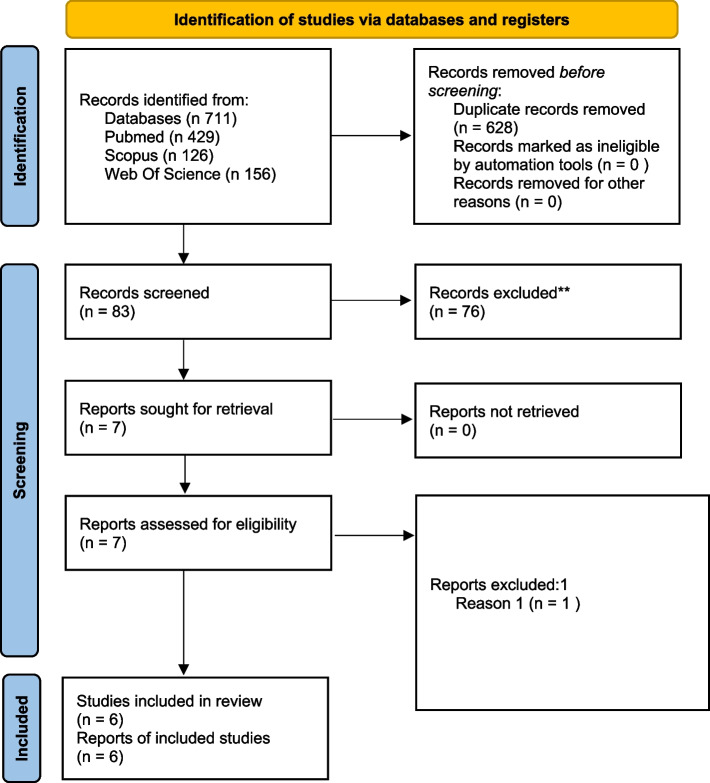
The Preferred Reporting Items for Systematic Reviews and Meta-Analyses 2020 flowchart used for the study selection

The searches of PubMed, Scopus, and Web of Science yielded 429, 126, and 156 articles, respectively, for a total of 711 records. Eighty-three articles were left after removal of 628 duplicates. After the initial screening (title and abstract), 76 articles were excluded. The seven articles left were subjected to a full-text review for eligibility assessment [18, 22–26, 45]. One article was excluded because it did not satisfy the inclusion criteria [40]. Finally, six articles were used for the systematic review [18, 22–26] (Fig. 1).

The following exclusion criteria were: [1] Studies where participants were not affected by AP, [2] ex vivo*, *in vitro*, and *in silico* models only* studies, [3] studies that included experimental models with comorbidities or with administration of other medications, [4] studies that did not provide a detailed description of the methodology used, [5] review papers, [6] opinion articles and [7] conference abstracts were excluded.

### Characteristics of the studies

The six in vivo studies selected were published between 2009 and 2018 [18, 22–26]. Among the included articles, only one was a retrospective human cohort study [18]. The present cohort study analyzed whether there was an association between statin intake and AP healing following NsRCT. All patients included in this investigation (30 cases and 30 controls) received a well-performed initial NsRCT or re-treatment in teeth presenting with AP lesions with a minimum diameter of 3 mm. The treatment was performed by the endodontic residences in a university setting from 2011 to 2014. Patients who took therapies that can alter bone metabolism were excluded, and all other confounding variables that may influence the outcome of endodontic treatment (age, sex, diabetes, smoking and cardiovascular diseases, periapical diagnosis, follow-up, primary or secondary root canal treatment, and tooth type) were considered in the multivariate logistic regression analysis. The patients were treated using different types of statins (*simvastatin*, *atorvastatin*, *pravastatin*, *rosuvastatin*, *lovastatin*) at different dosages [10, 20, 40, 80 mg daily], whereas the controls were thirty healthy subjects without AP. All patients meeting the inclusion criteria of the study had completed a follow-up examination 2–5 years post-treatment, including radiographic and clinical evaluation. AP healing was assessed using the periapical index (PAI) [46], which was attributed to the radiographs by two calibrated and expert endodontists [18].

Among the other five articles selected, one was an in vivo animal study [26], and all the others were combined in vitro*/*in vivo animal studies [22–25]; however, in respect of the inclusion criteria, only the in vivo experiments part of the research were considered. The animal species employed included *Sprague–Dawley* rats [22–25] and *Wistar* rats [26]. Four studies used a case sample of 10 rats compared to 10 controls [22–25], and the fifth study analyzed 25 rats: 12 cases, 12 controls, and 1 negative control (without any intervention) [26]. In all experiments, one mandibular molar from each rat was selected [22–26]. In three studies, AP was induced by accessing the pulp chamber and leaving it open in all treatments time [22–24]. Alternatively, AP was induced by extirpating the pulp, contaminating the canals with saliva, filling the access with temporary cement [26], or sealing the exposed pulp chambers with amalgam [25]. In all the included studies, the drug compound examined was *simvastatin,* which was administered to the animals by injections given either on days 1 and 7 [22, 23], at day 0 and then every 5 days until day 21 [24], or from day 20 every 5 days until day 35 [25], depending on the protocol. In one research, the rats were fed using gavage for 20 days, starting 10 days before pulp exposure [26]. The dose of simvastatin administered was 20 mg/kg in all the five animal studies, representing the equivalent of a human dose of about 1.6 mg/kg, which is a little higher than that used in a standard therapy for humans (0.1–1.0 mg/kg per day). Four studies used both immunohistochemistry and imaging, bidimensional radiographs [22–25], and computerized tomography (CT) [25] to investigate AP, whereas in one experiment, only immunohistochemical analysis was conducted [26].

In all these studies, the protective activity of statins against bone resorption resulting from the inflammatory processes of AP was evaluated. All the experiments included a study group (animals under the simvastatin effect) and a control group (animals receiving placebo). Osteoblasts are the most commonly considered cells. Lai et al. [23] and Yang et al*.* [25]*,* examined the importance of the balance of apoptosis/autophagy/mitophagy mechanisms of osteoblasts during the development of AP. Both studies evaluated the occurrence of apoptosis through a procedure called the terminal deoxynucleotidyl transferase-mediated deoxyuridine triphosphate nick end-labeling (TUNEL) reaction, which allows the detection of DNA strand breaks, which are the result of endonuclease activity during the late stage of the apoptotic cascade [23, 25]. Lai et al*.,* also considered the number of osteoblasts with Beclin-1 + , a marker of the autophagic process [23], whereas Yang et al. analyzed hypoxia-induced mitophagy in osteoblasts in the presence of PTEN-induced kinase 1 (PINK-1) in these cells in AP [25]. Shadmehr et al. focused on the expression of osteoprotegerin (OPG) and receptor activator of NF-kappa B LIGAND (RANK-L), fundamental for the regulation of bone apposition in AP, by means of RNA extraction and quantitative real-time PCR [26]. Finally, in two studies [22, 24] the recruitment of histiocytes/macrophages in the resorption lacunae of AP was evaluated by testing the presence of the inflammatory process Cluster of Differentiation 68 (CD68). Furthermore, they analyzed the expression of some inflammatory markers in osteoblasts, such as cysteine-rich angiogenic inducer 61 (Cyr 61), a TNFα-stimulated protein that promotes cell adhesion, chemotaxis, angiogenesis, and regulates tumor growth [22, 24], the chemokine C–C motif ligand 2 (CCL2), which is involved in the recruitment of monocytes, memory T cells, and dendritic cells to the sites of inflammation, and PHOSPHO-Forkhead box class O 3a (p-FoxO3a), an inactivated transcriptional repressor of Cyr61 by phosphorylation [24].

The treatment groups and experimental design employed in each of the included studies are described in detail in Table 1.

**Table 1:** Characteristics of the studies included (in chronological order)

Author and year	Strain (Species)	Sample	Disease induction	Statins	
Lin et al. 2009 [22]	Sprague Dawley rats	20 (10–10)	Pulpal exposure performed at the distal fossa of right mandibular first molars	*Simvastatin*	
Lai et al. 2012 [23]	Sprague–Dawley rats	20 (10–10)	Pulpal exposure performed at the distal fossa of right mandibular first molars	*Simvastatin*	
Shadmehr et al. 2013 [26]	Wistar rats (Rattus *norvegicus albinus*)	25 (12–12-1)	Pulp of mandibular first molar was extirpated and the canal was contaminated with saliva. The cavities were filled with Cavit	*Simvastatin*	
Lin et al. 2013 [24]	Sprague–Dawley rats	20 (10–10)	Pulpal exposure performed at the distal fossa of right mandibular first molars and cavity was left open	*Simvastatin*	
Yang et al. 2019 [25]	Sprague–Dawley rats	20 (10–10)	Pulpal exposure performed at the distal fossa of right mandibular first molars and left open. On day 20, the coronal pulp chambers were cleansed and the access cavities sealed with amalgam	*Simvastatin*	
Alghofaily et al. 2018 [18]	Humans	60 (30–30)	AP already present (at least 3 mm)	*Simvastatin, atorvastatin, pravastatin, rosuvastatin, lovastatin*	
Author and year	Statins administration	Follow up	Outcome Measurements	Results	Source of funding
Lin et al. 2009 [22]	Subcutaneous injection at day -1 and at day 7	20 days	Radiological, Immunohistochemical and Histological analysis (H&E)	Simvastatin diminished periapical bone destruction. Only a few Cyr61 + and CD68 + osteoblasts were detected in simvastatin treated animals	Grants NSC96-2314-B002-180-MY3 and NTUH98-S-1149 from the National Science Council, Taiwan and National Taiwan University Hospital
Lai et al. 2012 [2012] [23]	Subcutaneous injection at day -1 and at day 7	20 days	Radiological and Immunohistochemical analysis	Simvastatin diminished periapical bone destruction and reduced the number of apoptotic osteoblasts and the extension of periapical lesions in rats. The number of Beclin-1-synthesizing osteoblasts also increased after simvastatin treatment	No source of funding has been declared in the study
Shadmehr et al. 2013 [26]	Gavage feeding from 3 days before the pulp exposure for 10 days	1,2 or 4 weeks	RNA extraction Quantitative Real Time PCR	Simvastatin influence the expression of OPG and RANKL genes in a time dependent manner	Funding of Vice Chancellery for Research, Isfahan University of Medical Sciences (389,217)
Lin et al. 2013 [24]	Subcutaneous injection at day 0 and then every 5 days till the 21st day	21 days	Radiological and Immunohistochemical analysis	Simvastatin diminished periapical bone destruction. oCyr61 + , p-Fox3a + , CCL2 + osteoblasts and CD68 + macrophages were significantly lower in simvastatin treated animals	No source of funding has been declared in the study
Yang et al. 2019 [25]	Subcutaneous injection from day 20 and then every 5 days till the 35st day	35 days	Radiological and Immunohistochemical analysis	Simvastatin reduces periapical bone resorption, considering size and volume. A few PINK1 + and TUNEL + osteoblast were detected in simvastatin treated animals	Grants to SHK from Ministry of Science and Technology of Taiwan (MOST103-2314-B-002–094-MY2; MOST 105–2314-B-002–076; MOST 106–2314-B-002–026-MY3) and National Taiwan University Hospital (NTUH105-S3016; NTUH106-S3422), SKL from Ministry of Science and Technology of Taiwan (MOST104-2314-B-002–154-MY3) and National Taiwan University Hospital (NTUH106-S3489; NTUH 105-S3076)
Alghofaily et al. 2018 [18]	10,20,40,80 mg daily per os	2–5 yrs	Periapical index score (PAI) and clinical examination	The healing outcome was higher for the statin group compared with the control group	No source of funding has been declared in the study

*AP* Apical Periodontitis, *H&E* haematoxylin and eosin

### Quality assessment

**Fig. 2:**
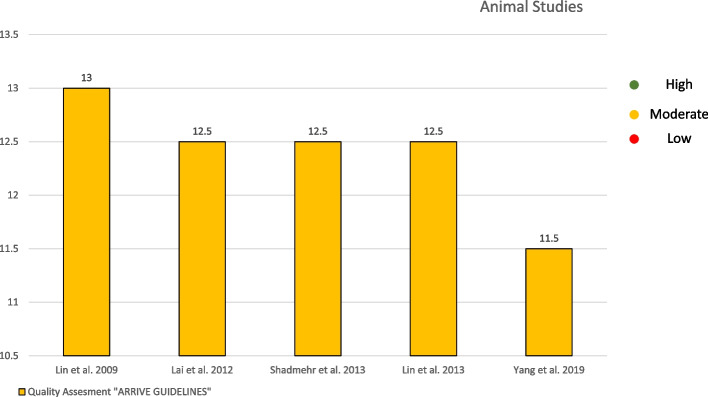
Quality assessment of the included studies on animals following the animal research: reporting of in vivo experiments guidelines and the systematic review centre for laboratory animal

**Fig. 3:**
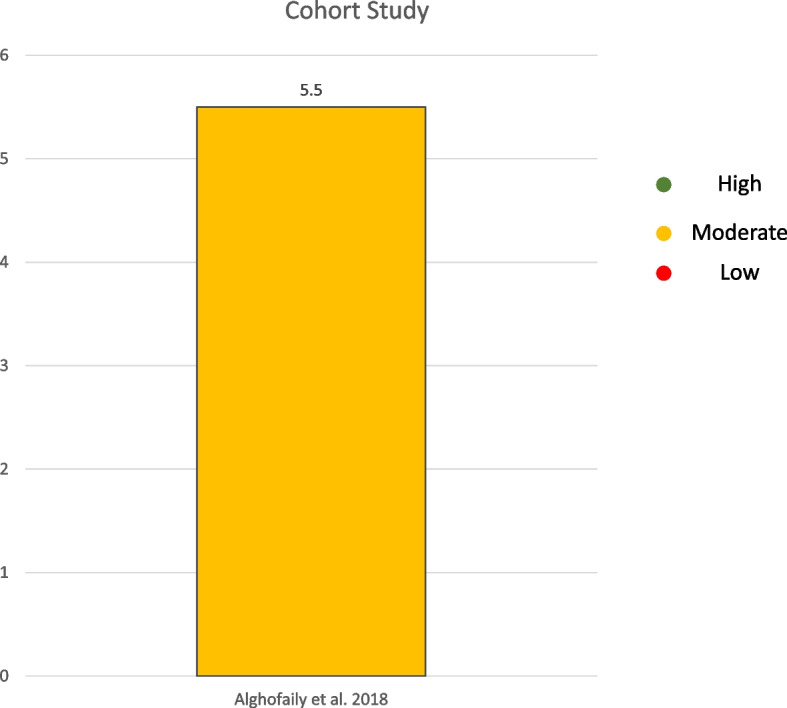
Quality assessment of the Cohort Study included following the Newcastle–Ottawa Quality Assessment Form. For Cohort Studies guidelines and the Tool to assess risk of bias in cohort studies of CLARITY Group

The five animal studies evaluated using the ARRIVE guidelines showed a moderate grade of quality [22–26] (Fig. 2). The cohort study was evaluated using the “Newcastle Ottawa Quality Assessment Form for Cohort Studies” guidelines and exhibited a moderate grade of quality [18] (Fig. 3).

### Risk of bias

**Fig. 4:**
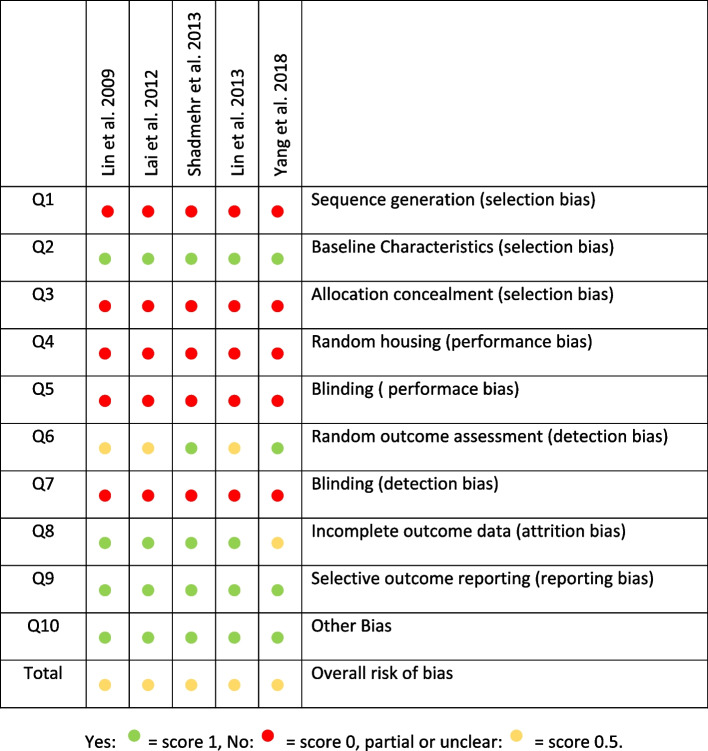
Risk of bias of the included studies on animals following the Animal Research: Reporting of In Vivo Experiments guidelines and the Systematic Review Centre for Laboratory animal Experimentation RoB tool

Five animal studies evaluated using the SYRCLE tool presented a moderate risk of bias [22–26] (Fig. 4).

**Fig. 5:**
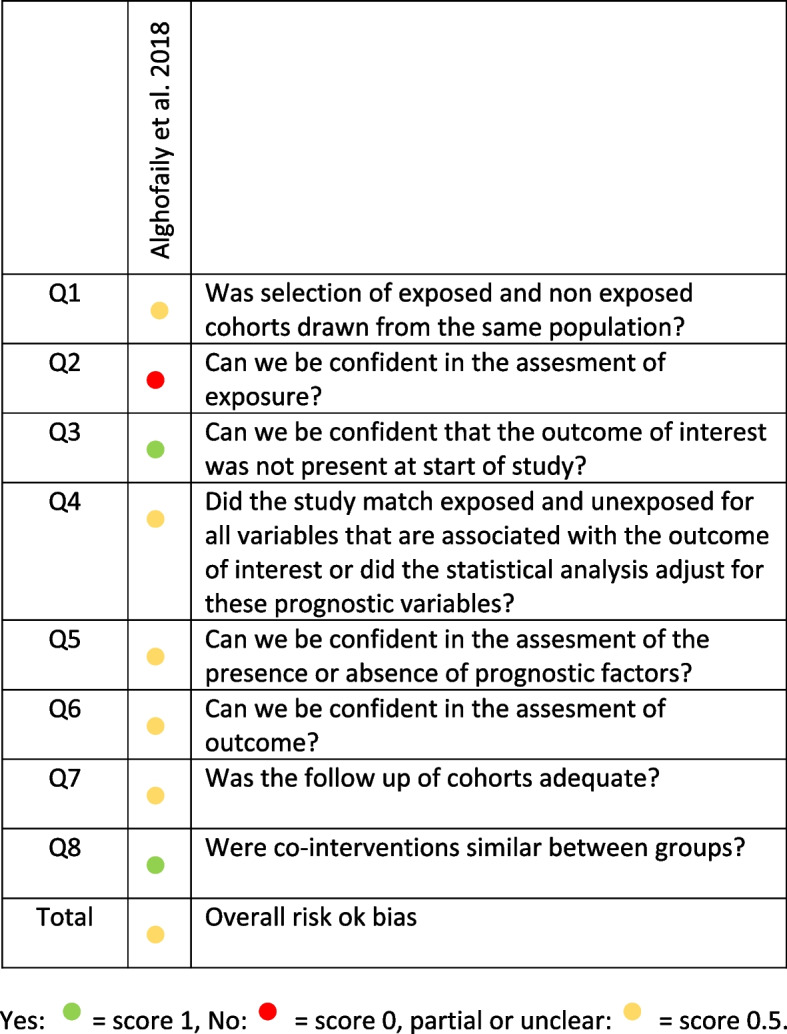
Risk of bias of the cohort study included following the tool to assess risk of bias in cohort study of CLARITY group

The cohort study was evaluated with the “Tool to assess risk of bias in cohort studies of CLARITY Group,” and it also showed a moderate risk of bias [18] (Fig. 5).

The eligible studies showed a moderate overall quality and a moderate risk of bias. Considering the scarcity of studies investigating this topic, all studies meeting the inclusion criteria were included in this review to provide the best and most comprehensive available evidence.

### Answer to the focused question

*Can systemic administration of statins affect the inception and healing of AP in human and animal models?*

All included studies showed a beneficial effect of statins on AP in humans and experimental animal models (Table 1) [18, 22–26].

The hypothesis that statins could be associated with an increased healing rate of AP was supported by the retrospective cohort study in humans [18]. The rate of healing of AP in 30 patients undergoing treatment with statins, at 2 to 5 years follow-up was significantly higher in the study group (93%) compared to that in the control (70%), even though the cases were older in age than the controls (63 ± 9.7 *vs* 53.8 ± 16.1 years), and the patients were prescribed different types and dosages of statins. It is important to underline that this altered match between cases and controls, which make the results even more significant, can also be considered the main limitation of this included study.

In animal research, *simvastatin* appeared to positively affect the development of induced AP. In most studies [22–26], the radiographic [22–24], tomographic [25], and image analyses [22–25] showed that bone resorption was significantly attenuated in animals which were administered subcutaneous injections of simvastatin, either immediately before [22, 23], at the same time [24], or after [25] the induction of AP. This effect was observed in the average sizes [22–24] and volumes [25] of the AP lesions, which were significantly smaller in the animals treated with statins than in the controls.

Better preservation of bone integrity, manifested as smoother bony contours in the periradicular lesions of the involved teeth, was also observed in the research group as a possible result of a less aggressive osteolytic activity occurring in the lesions [23, 24]. Furthermore, when the most important factors involved in osteoclast differentiation were analyzed [26], the expression of RANKL decreased, while OPG, an inhibitor of the resorptive effects of RANKL, increased in a time-dependent manner in the *simvastatin* group [26]. Lastly, immunohistochemistry showed that mitophagy (PINK-1) [25], apoptosis (TUNEL) [23, 25], and pro-inflammatory markers (Cyr-61, CCL2, p-FoxO3a, and CD68) [22, 24] were significantly lower in the statin-treated rats. The only autophagy marker considered (Beclin-1) was prominent in osteoblasts and fibroblasts of the study group, suggesting that autophagy exerts a protective role against cell death during the development of AP [23]. These data demonstrate that there is an association between statin administration and protective action during the inception of AP in animal models.

## Discussion

Apical periodontitis is a disease characterized by a prevalently chronic inflammatory response to infection of the root canal system of the affected tooth and is usually represented by the presence of a radio-transparent lesion located around the apical portion of the root [1–3].

The pathogenesis, development, and healing of AP are regulated by intertwining immunological mechanisms that influence bone destruction and apposition [1–3, 9, 16, 18–20, 47]. Apical periodontitis is a highly prevalent disease involving 52% of the adult population worldwide, and noticeably, it is more frequently found in teeth that have already undergone NsRCT [48]. These data pose further concerns regarding the healing of AP following treatment, as the presence of untreated or persistent AP represents both an inflammatory burden and a factor that ultimately leads to loss of teeth, which in turn is associated with a lower quality of life of the older population [49]. The treatment for the resolution of AP is based on the elimination or at least the reduction of the microbial load from the root canal through the accurate instrumentation, disinfection and obturation of the endodontic space, followed by an adequate coronal restoration of the tooth to prevent re-infection [50].

Following NsRCT, the potential for a favorable outcome of AP is in the range of 75–80% [6], and the volume of untreated canals is significantly associated with the persistence of the disease [51]. The healing of AP is also influenced by host health conditions [10] and by their predisposition to stronger inflammatory reactions [11, 12]. Based on these concepts, there has been growing attention on how intracanal medications and various drugs taken systemically may affect the healing of AP, possibly by attenuating the inflammation caused by endodontic infection [13, 14, 19, 20, 52, 53], or promoting its resolution after treatment [15, 18].

The main limit of this systematic review is related to its qualitative and descriptive nature, and to the impossibility to produce a meta-analysis. Furthermore, almost all data are based on animal studies and it is important to emphasize that the translation of the results obtained in the included studies to humanreactions is limited. The review aimed to analyze the role of systemic intake of statins in the development and healing of AP, as these medications, in addition to their effect on cholesterolemia, possess antibacterial [54], anti-inflammatory, and immunomodulatory action [29–31]. Of the six articles investigated (Table 1), only the retrospective cohort study found a significant relationship between statin intake and the rate of healing of AP at 2- to 5- year follow-up and indicated that statins contributed to a 10% increase in healed periapical lesions associated with NsRCT [18]. This result seems to be even more interesting when considering that the patients in the study group were older than the controls and that they were taking different kinds of statins at various dosages, *simvastin* being the most used. At the same time the difference in the age of the two groups and the variety of statins and dosages assumed by the patients in this study, represent also its major limitation, associated to the small sample size.

Beneficial effects of statin intake on AP were also consistently shown in all the selected in vivo studies conducted in experimental animal models [22–26], in which the objective was to observe whether the administration of statins could influence the inception and development of AP. In all these studies, *simvastatin* appeared to affect the development of induced AP, because the animals that were administered the medications using different protocols (Table 1) had significantly reduced lesion size (61.3% to 57.4%) [22–24] and volume [25] compared to the controls. The lesions also exhibited more regular bone contours, hypothetically as the result of a milder osteolytic activity [23, 24], which in turn was explained by the decreased expression of RANKL and the parallel increase in the expression of OPG [23, 26], and by the lower concentration of mitophagy [25], apoptosis [23, 25], and pro-inflammatory markers [22, 23] found in statin-treated animals. These results are thus promising, even if they need to be interpreted with caution because obtained from animal models, and supported by laboratory experiments.

However, when adjunctive intake of statins was considered with regard to periodontal therapy, it resulted in better clinical and radiographic parameters, but the evidence was considered too low to conclude that these medications enhance periodontal treatment [37, 55].

Last, even though statins are considered safe and well tolerated, they could determine few side effects [56], the most severe of which is myotoxicity in its various forms, including myopathy, myalgia, myositis and rhabdomyolysis [57]. These adverse conditions are usually related to drug interactions (60% of cases) [58] and to high dosage chronic treatments which could led to cellular membrane hyperexcitability [59].

## Conclusions

Within the limitations of the reported studies and of this systematic review, it can be observed that statins may have a valuable impact on the health of the periapical and periodontal tissues, which could be attributed to their anti-inflammatory properties and immunomodulatory effects, which could prevent the development of extensive lesions of AP and positively influence their resolution upon treatment. In light of these conclusions, well-designed clinical studies on humans are required to confirm the effects of these medications. An investigation on the prevalence of apical periodontitis in patients taking statins could give further information on the effect of statins on AP. Then, testing a topical application of statins within a formula for an intracanal medication or inside a root canal sealer could be evaluated.

### Supplementary Information

**Additional file 1:** **Supplementary table 1.** Prisma 2020 checklist for abstracts.

**Additional file 2:** **Supplementary table 2.** Prisma 2020 Checklist.

**Additional file 3: Supplementary Table 3. **Details of the number of articles retrieved from each database and search strategy.

## Data Availability

The datasets used and/or analysed during the current study available from the corresponding author on reasonable request.
