# Supplementary material for: The role of systemic statins in the inception and healing of apical periodontitis: a systematic review
Source: BMC Oral Health. 2023 Oct 7;23:730. doi: 10.1186/s12903-023-03472-3 (PMC10560424; doi:10.1186/s12903-023-03472-3)
Supplement: Supplementary file 3 — Additional file 3: Supplementary Table 3. Details of the number of articles retrieved from each database and search strategy. [file 12903_2023_3472_MOESM3_ESM.docx]

**Supplementary Table 3.** Details of the number of articles retrieved from each database and search strategy

| Block 1 |  | PubMed | Scopus | Word of Science | All Search Engines |
| --- | --- | --- | --- | --- | --- |
| statin apical periodontitis | | 12 | 5 | 4 |  |
| statin endodontic treatment | | 27 | 4 | 4 |  |
| statin endodontic lesion | | 2 | 1 | 3 |  |
| statin endodontic | | 40 | 5 | 4 |  |
| statin endodontics | | 40 | 4 | 6 |  |
| statin root canal treatment | | 12 | 4 | 2 |  |
| statin endodontic diseases | | 11 | 5 | 3 |  |
| statin periapical lesion | | 5 | 2 | 6 |  |
| statin pulp healing | | 5 | 2 | 1 |  |
|  |  |  |  |  |  |
| Total Block 1 |  | 154 | 32 | 33 | 219 |
| Block 2 |  |  |  |  |  |
| simvastatin apical periodontitis | | 6 | 7 | 10 |  |
| simvastatin endodontic treatment | | 22 | 7 | 8 |  |
| simvastatin endodontic lesion | | 3 | 3 | 5 |  |
| simvastatin endodontic | | 34 | 12 | 10 |  |
| simvastatin endodontics | | 34 | 7 | 15 |  |
| simvastatin root canal treatment | | 9 | 9 | 4 |  |
| simvastatin endodontic diseases | | 5 | 4 | 4 |  |
| simvastatin periapical lesion | | 6 | 8 | 13 |  |
| simvastatin pulp healing | | 2 | 5 | 4 |  |
|  |  |  |  |  |  |
| Total Block 2 |  | 121 | 62 | 73 | 256 |
| Block 3 |  |  |  |  |  |
| statins apical periodontitis | | 12 | 5 | 8 |  |
| statins endodontic treatment | | 27 | 4 | 4 |  |
| statins endodontic lesion | | 2 | 1 | 4 |  |
| statins endodontic | | 40 | 5 | 5 |  |
| statins endodontics | | 40 | 4 | 11 |  |
| statins root canal treatment | | 12 | 4 | 5 |  |
| statins endodontic diseases | | 11 | 5 | 3 |  |
| statins periapical lesion | | 5 | 2 | 9 |  |
| statins pulp healing | | 5 | 2 | 1 |  |
|  |  |  |  |  |  |
| Total Block 3 |  | 154 | 32 | 50 | 236 |
| Total of Block 1, Block 2 and Block3 |  | 429 | 126 | 156 | 711 |

|  | PubMed | Scopus | Web of Science | All Search Engines |
| --- | --- | --- | --- | --- |
| Total of Block 1, Block 2 and Block3 | 429 | 126 | 156 | 711 |
| Copies | 371 | 88 | 123 | 628 |
| Articles | 58 | 38 | 33 | 83 |
